# The Effect of Oral Supplementation with a Multi-Strain Probiotic Preparation on Group B Streptococcus (GBS) Carriage in Pregnant Women—A Pilot Study

**DOI:** 10.3390/jcm15031113

**Published:** 2026-01-30

**Authors:** Katarzyna Zych-Krekora, Oskar Sylwestrzak, Michał Krekora

**Affiliations:** 1 Department of Perinatology, Obstetrics and Gynecology, Polish Mother’s Memorial Hospital Research Institute, 93-338 Lodz, Poland; 2 Department of Obstetrics and Gynecology, Polish Mother’s Memorial Hospital Research Institute, 93-338 Lodz, Poland; 3 Department of Prenatal Cardiology, Polish Mother’s Memorial Hospital Research Institute, 93-338 Lodz, Poland; krekoram@poczta.onet.pl

**Keywords:** Group B streptococcus, pregnancy, rectovaginal carriage, probiotics, *Lactobacillus crispatus*, pilot study, microbiota modulation

## Abstract

**Background/Objectives**: Maternal rectovaginal carriage of Group B Streptococcus (GBS, *Streptococcus agalactiae*) is a major risk factor for vertical transmission and early-onset neonatal infection. Intrapartum antibiotic prophylaxis reduces early-onset disease but does not address antenatal carriage and may affect the maternal–neonatal microbiota. Microbiota-directed interventions, including probiotics, are being explored as complementary strategies. **Methods**: This prospective, single-centre, open-label pilot intervention study included 10 pregnant women (18–40 years) with singleton pregnancies and a positive vaginal and/or rectal GBS swab, without pre-gestational or gestational diabetes and without antibiotic use in the 4 weeks before enrolment. Participants received OMNi-BiOTiC^®^ FLORA plus (multi-strain lactic acid bacteria, including *Lactobacillus crispatus*) orally at 2 × 2 g/day from the 15th to the 34th gestational week. Microbiological swabs were obtained at qualification (12–15 weeks), mid-pregnancy (22–25 weeks), and late pregnancy (34–35 weeks). Outcomes were described descriptively. **Results**: Among 56 screened pregnant women, 10 were GBS-positive (17.9%) and enrolled. All participants were GBS-positive at baseline. At 22–25 weeks, 5/10 (50%) had a negative GBS result. At 34–35 weeks, 9/10 (90%) were GBS-negative, while 1/10 (10%) remained colonised. Time to first negative result ranged from 7.6 to 20.2 weeks from supplementation start (median 8.6 weeks). No recurrences (negative-to-positive transitions) were observed between the second and third sampling points. No adverse events related to supplementation were reported. In contrast, among the 46 women who were GBS-negative at screening and did not receive probiotic supplementation, 14 (30.4%) were found to be GBS-positive at routine screening performed at 35–37 weeks of gestation. **Conclusions**: In this pilot single-arm study, oral supplementation with a multi-strain probiotic preparation during pregnancy was associated with a time-dependent reduction in rectovaginal GBS carriage and was well tolerated. These preliminary findings support the feasibility of larger randomised controlled trials incorporating microbiome profiling and neonatal outcomes.

## 1. Introduction

Group B streptococci (Streptococcus agalactiae, GBS) are an important perinatal pathogen of global significance, often colonising the rectal and vaginal areas of pregnant women without clinical symptoms [1].

It is estimated that approximately 19.7 million pregnant women worldwide are carriers of GBS, corresponding to an average colonisation rate of approximately 17.1% [2]. However, the frequency of colonisation varies considerably by region, ranging from 10% to over 30%, depending on the population studied, the diagnostic methods used and the sampling strategies [1,3].

GBS colonisation is dynamic and may change during pregnancy, with some women transitioning from colonised to non-colonised status. Approximately 18–20% of pregnant women experience transient colonisation, while 20–30% experience persistent colonisation, which is associated with a continuous risk of vertical transmission [4,5,6,7]. The main reservoir of GBS remains the recto-vaginal area, from which colonisation of the vagina and rectum occurs, which is crucial for the transmission of bacteria to the newborn [8]. Vertical transmission of GBS can occur both prenatally, as a result of ascending infection from the genital tract, and, much more frequently, during delivery, when the newborn comes into contact with the colonised birth canal [9]. GBS infection in newborns is associated with serious clinical consequences and includes an early form of the disease, occurring in the first week of life, most often manifested by sepsis, pneumonia or meningitis, and a late form of the disease, developing between the 7th day and the 3rd month of life, in which meningitis is more common [10]. Streptococcus agalactiae colonisation in pregnant women is also associated with adverse obstetric outcomes such as preterm birth, miscarriage and chorioamnionitis [11,12]. The consequences of these infections can have a long-term impact on the health of the child, including an increased risk of neurodevelopmental disorders, chronic respiratory diseases, and metabolic and cardiovascular diseases [13]. The importance of GBS epidemiology therefore extends beyond the neonatal period and highlights the need for effective prevention and treatment strategies targeting both maternal and infant health [14].

A single screening test may not reflect the actual risk of vertical transmission, which justifies consideration of standardising diagnostic methods, including simultaneous vaginal and rectal swabs, which are more sensitive in detecting colonisation [15,16,17].

The dynamics of colonisation are also influenced by the diversity of GBS serotypes circulating in the population, as some serotypes show a greater ability to adhere, form biofilms and persist in the host organism [18,19]. Maternal factors such as age, ethnicity, obesity, and environmental and hygiene conditions are also important [18,20]. The asymptomatic nature of colonisation further complicates the assessment of its actual frequency [1].

GBS colonisation rates show considerable geographical variation [9,21]. These discrepancies largely reflect differences in diagnostic methods, sampling strategies and the characteristics of the populations studied [22,23,24,25].

Asian populations generally show lower colonisation rates, estimated at 5–10%, although values ranging from 0.3 to 5.9% have been reported in individual regions of East Asia [26,27]. In contrast, some African populations have been found to have exceptionally high colonisation rates exceeding 30%, which may partly explain the increased risk of intrauterine GBS transmission observed in newborns of African descent [28,29]. Ethnicity also remains an important risk factor, with studies indicating a higher risk of early-onset neonatal sepsis in children of African American mothers colonised by GBS [28].

The variability in the frequency of GBS colonisation is due not only to genetic, environmental and socio-economic factors, but also to significant methodological differences between studies [30,31]. The lack of standardisation of diagnostic methods and surveillance makes it very difficult to directly compare epidemiological data between regions and to accurately estimate the global burden of GBS disease [32].

An additional challenge is the dynamic nature of GBS colonisation, including transient and sporadic carriage during pregnancy, which limits the value of a single screening test in assessing the risk of vertical transmission. This phenomenon highlights the importance of longitudinal studies and the use of molecular typing methods to better understand the epidemiology of GBS colonisation and its impact on perinatal outcomes [33,34].

Although molecular methods enable faster and more sensitive detection of GBS, their routine use remains limited due to cost and infrastructure requirements, particularly in resource-constrained countries [35,36,37].

In response to these challenges, research is ongoing to develop rapid, cost-effective, and highly sensitive point-of-care diagnostic methods to improve the identification of GBS colonisation and perinatal outcomes [38]. Advances in molecular diagnostics enable faster and more accurate detection of GBS, but their routine clinical use remains limited due to cost, infrastructure requirements, and difficulties in assessing the clinical significance of detecting genetic material that does not always reflect active colonisation or infection [39,40,41].

An additional challenge to current prevention strategies is the growing resistance to antibiotics, which may limit the effectiveness of antibiotic prophylaxis during delivery and highlights the need to develop alternative methods of preventing GBS infections [38,42]. In this context, immunoprophylactic strategies, including maternal vaccination against GBS, are of particular interest as a potential method of reducing the incidence of early-onset GBS disease by inducing high concentrations of serotype-specific antibodies in pregnant women [43].

The development of a vaccine providing broad protection remains a major challenge due to the high antigenic diversity of the GBS capsular polysaccharide, which includes at least ten serotypes [44]. Current clinical trials include conjugate vaccines targeting capsular polysaccharides and protein vaccines targeting conserved surface antigens, evaluated for their effectiveness in preventing GBS infections in newborns and pregnant women [45,46].

The importance of these strategies is particularly relevant in light of the limitations of antibiotic prophylaxis during labour, which effectively reduces the incidence of early-onset GBS disease but does not prevent late-onset disease and may contribute to the selection of resistant strains [17,47]. Data suggest that maternal vaccination has the potential to provide protection against both early-onset and late-onset GBS disease, offering a more comprehensive approach to prevention [48].

Identification of specific serotypes and sequence types of Streptococcus agalactiae that are more commonly associated with invasive disease and antibiotic resistance is critical for understanding the pathogenesis of GBS and for developing vaccines and surveillance programmes targeted at high-risk strains [42,49,50,51].

**Mechanisms of vertical transmission and consequences for newborns**

Vertical transmission of Streptococcus agalactiae (GBS) from an infected mother to her newborn can occur in two main ways, antepartum and intrapartum, each of which is associated with different risks of GBS disease in the newborn [52]. Antepartum infection, although less common, can take the form of ascending infection and lead to preterm labour, premature rupture of membranes, and congenital GBS infection [53,54].

The risk of intrauterine infection is primarily influenced by the high degree of GBS colonisation in the mother’s urogenital tract and the duration of membrane rupture, as well as the gestational age and immune status of the newborn [24,55]. The clinical picture of GBS infection in newborns ranges from asymptomatic bacteraemia to severe forms of the disease, such as sepsis, pneumonia and meningitis. The mortality rate among affected infants is 10–50%, and surviving children often experience long-term neurological sequelae, including hearing and visual impairment, developmental delays, and cerebral palsy [56,57].

It is estimated that in 2020, GBS colonisation affected approximately 20 million pregnant women worldwide, resulting in nearly 400,000 cases of early-onset or late-onset GBS disease in newborns and approximately 90,000 infant deaths [58].

**The importance of antibiotic prophylaxis during labour**

The key role of antibiotic prophylaxis during labour (IAP) in reducing the incidence of early-onset GBS disease in newborns is well documented [42,59,60].

The implementation of universal screening strategies for GBS colonisation and targeted antibiotic use during labour has become the cornerstone of modern obstetric practice. In countries where antibiotic prophylaxis is routinely used, the incidence of early-onset GBS disease has decreased by approximately 80% [25,61,62].

According to current CDC guidelines, GBS screening is performed at 36 0/7–37 6/7 weeks of gestation, and perinatal antibiotic therapy is used in colonised women [63,64,65,66,67,68,69,70].

**Antibiotic resistance and IAP limitations**

Penicillin G and ampicillin are most commonly used in IAP prophylaxis [71,72]. Cefazolin is recommended for women who are allergic to penicillin [21,23].

Although GBS resistance to penicillin remains relatively low, there is an increasing decline in sensitivity to β-lactam antibiotics and a high level of resistance to macrolides and lincosamides [22,42,50,73,74].

Growing antibiotic resistance, combined with the limited effectiveness of IAP in preventing late-onset GBS disease, highlights the need to develop complementary strategies [17,19,20,50,75,76,77,78].

An additional barrier is implementation issues, particularly in resource-limited settings, where access to antenatal care and the logistics of immunisation programmes may limit the effectiveness of population-based interventions [9,79]. There is growing interest in interventions aimed at modulating the vaginal and intestinal microflora of pregnant women, including the use of microflora preparations that may influence GBS colonisation and the risk of obstetric complications [80,81]. This approach is also being considered in the context of reducing the adverse effects of widespread antibiotic use, including resistance selection and microflora disturbances in newborns [82,83].

**Vaginal microbiota during pregnancy**

The vaginal microbiota undergoes significant physiological changes during pregnancy, which primarily include a reduction in the diversity of microorganisms and the dominance of bacteria of the genus *Lactobacillus*, in particular *Lactobacillus crispatus* [83,84]. This microflora composition helps to maintain a low vaginal pH, which creates an environment unfavourable for the growth of pathogenic bacteria, including Streptococcus agalactiae (GBS) [81,84]. Maintaining eubiosis dominated by *L. crispatus* is inversely correlated with GBS colonisation, indicating its important protective role in preventing infection and vertical transmission [80,81].

A reduction in Lactobacillus bacteria, especially *L. crispatus*, and the accompanying increase in microbial diversity is associated with an increased risk of GBS colonisation and adverse pregnancy outcomes [15,81,85]. Such microflora disturbances may weaken the protective barrier of the vaginal epithelium, increasing susceptibility to ascending infections and obstetric complications, including preterm birth [15,81,85]. Dysbiosis characterised by a decrease in the number of lactic acid-producing bacteria leads to an increase in vaginal pH, which promotes the proliferation of GBS and other potential pathogens [81,83,84].

However, the protective importance of the vaginal microflora is not the same for all Lactobacillus species. In particular, Lactobacillus crispatus exhibits stronger protective properties compared to other species such as Lactobacillus iners [84,86,87]. The dominance of *L. crispatus* is consistently associated with a lower risk of GBS colonisation, bacterial vaginosis and preterm birth, while the predominance of *L. iners* is more commonly observed in transient and dysbiotic states [86,87].

The protective mechanisms of *L. crispatus* primarily involve the production of lactic acid and the maintenance of a low vaginal pH, which inhibits the growth of pathogens [84]. In addition, *Lactobacillus* bacteria can produce antibacterial factors (e.g., bacteriocins) that limit the growth of pathogens, including GBS [81,84].

The instability of microflora dominated by *L. iners* promotes a transition to more complex and dysbiotic microbial structures with a higher proportion of anaerobic bacteria [87], which is associated with a higher risk of microflora disturbances and adverse pregnancy outcomes [83,86]. The diversity of protective properties of individual Lactobacillus species highlights the importance of identifying microbiota at the species level in assessing vaginal health and the risk of GBS colonisation [15,81,83,84,85,86,87].

The functional diversity of individual Lactobacillus species clearly indicates the need to identify the vaginal microflora at the species level, rather than just the genus level, especially in the context of pregnancy. The presence of specific species, such as Lactobacillus crispatus, can significantly reduce the risk of GBS colonisation and affect the course of pregnancy, providing a basis for the development of targeted prevention strategies and microbiota interventions [79,88].

**Microflora composition and susceptibility to GBS colonisation**

The relationship between microflora composition and GBS carriage is confirmed by studies comparing the microbiological profiles of women with positive and negative GBS results. In women with a positive result, a more frequent co-occurrence of Gardnerella vaginalis and a lower presence of Lactobacillus jensenii were observed, suggesting a specific microbiological ‘signature’ associated with increased susceptibility to GBS [2]. In addition, GBS colonisation is sometimes associated with an increase in the abundance of other taxa, such as *Ureaplasma*, other *Streptococcus* species (other than GBS) and *Corynebacterium*, with a simultaneous decrease in the abundance of selected *Lactobacillus* species [2]. These results indicate that GBS carriage is more common in a vaginal microenvironment where the dominance of lactic acid-producing bacteria is weakened and, at the same time, the proportion of microorganisms typical of dysbiosis increases.

From the point of view of colonisation biology, both the competition between microorganisms for resources and the influence of the microflora composition on the parameters of the vaginal microenvironment are important. It is worth noting that *L. iners* and GBS share an auxotrophic dependence on many amino acids, which may intensify competition for limited nutrients and influence their mutual dynamics in the vaginal environment [2]. It should also be noted that greater heterogeneity of microbial profiles is described in GBS-positive groups. This may suggest that GBS colonisation is not only a consequence of dysbiosis, but may also potentially contribute to the destabilisation of the vaginal microflora, facilitating the growth of opportunistic microorganisms or weakening the host’s defence mechanisms [2].

In clinical practice, the inverse relationship between the abundance of lactic acid bacteria (especially a profile dominated by *L. crispatus*) and the risk of GBS carriage remains particularly important, as the acidic environment and antibacterial substances produced by *Lactobacillus* constitute a natural barrier to colonisation [2].

It is also worth noting that although the dominance of *Lactobacillus bacteria* as a genus is generally associated with a lower incidence of GBS carriage, this does not fully explain the differences in colonisation between women, suggesting the involvement of additional factors, both microbiological (presence of specific accompanying taxa, stability of the microbiota profile) and host-related (local immune response, behavioural and environmental factors) [1]. This caveat is important from an intervention design perspective: simply ‘increasing Lactobacillus’ may not be sufficient if species and functional levels are not taken into account.

Given the limitations of antibiotic prophylaxis during delivery and growing concerns about antimicrobial resistance, there is growing interest in microbiota interventions, particularly those based on the use of probiotics, as a potential alternative or complement to existing strategies for preventing *Streptococcus agalactiae* (GBS) during pregnancy.

This approach is part of a broader paradigm of host-directed prophylaxis, which aims to modulate the vaginal microenvironment towards stable eubiosis, rather than simply eliminating the pathogen [6].

The mechanisms by which Lactobacillus limit GBS colonisation are multifactorial and are not limited to the production of hydrogen peroxide [79]. Lactic acid production is crucial because it lowers the vaginal pH and creates an environment unfavourable for GBS growth [82,84]. Acidification of the microenvironment, combined with competition for nutrients and adhesion sites, is the main mechanism limiting pathogen colonisation [79,82,84].

In addition, individual Lactobacillus strains exhibit the ability to synthesise

- Bacteriocins with direct antibacterial activity;
- Biosurfactants that inhibit GBS adhesion to epithelial cells;
- Compounds that strengthen the epithelial barrier, which together contribute to the formation of a multi-layered defence system [79].

*Lactobacillus crispatus* occupies a special place among probiotics because it is highly stable in the vaginal microbiome and has strong antagonistic potential against pathogens (including GBS), and its functional profile is associated, among other things, with the presence of D-lactic acid isomer [82]. Unlike *L. iners*, which has lower stability and limited protective activity, *L. crispatus* has a more ‘protective’ metabolic profile that promotes the maintenance of eubiosis [82].

**Routes of administration and safety of probiotics**

Probiotics can be administered both orally and vaginally, with both routes of administration capable of modulating the vaginal microflora [80]. Vaginal preparations based on *L. crispatus* have been shown to effectively reduce the recurrence of bacterial vaginosis and help restore a Lactobacillus-dominated microflora profile.

Oral administration, although less direct, can affect the vaginal microbiome via the gut-vagina axis and is considered an alternative to local interventions [8,80].

## 2. Materials and Methods

**Hypothesis**

Supplementation with a multi-strain probiotic preparation containing selected strains of lactic acid bacteria, including *Lactobacillus crispatus* and, during pregnancy promotes beneficial modulation of the vaginal and rectal microbiota and leads to a reduction in Group B Streptococcus (GBS) colonisation.

**Main objective**

To evaluate the effectiveness of supplementation with a multi-strain probiotic preparation in modulating the vaginal and rectal microbiota and reducing Group B Streptococcus (GBS) carriage in pregnant women.

**Specific objectives**

1. To evaluate changes in the frequency of GBS colonisation of the vagina and rectum during supplementation with a multi-strain probiotic preparation.
2. To determine the minimum time required to achieve a reduction or eradication of GBS carriage after the start of supplementation.
3. To assess the safety and tolerability of supplementation with a multi-strain probiotic preparation in pregnant women.

**Type of study**

The study was a prospective, pilot, single-centre, open-label intervention study. It was designed to evaluate the effectiveness of supplementation with a multi-strain probiotic preparation in reducing Group B Streptococcus (GBS) carriage in pregnant women.

**Study population**

Ten pregnant womenwho met the eligibility criteria were included in the study. Participants were recruited from among patients attending routine obstetric visits at a single medical centre. All women provided written informed consentto participate in the study.

**Inclusion criteria**

Women who met the following conditions were included in the study:

- Aged 18–40 years;
- Single pregnancy between 15 and 34 weeks of gestation;
- Positive microbiological test result for GBS in a vaginal and/or rectal swab;
- No pre-gestational diabetes or gestational diabetes;
- No antibiotic therapy in the 4 weeks prior to inclusion in the study;
- Informed consent to participate in the study.

**Exclusion criteria**

The following women were excluded from the study:

- With multiple pregnancies;
- With significant chronic diseases that could affect the course of pregnancy or microbiota;
- With hypersensitivity or allergy to the ingredients of the probiotic preparation;
- Using other probiotics or antibiotics during the study.

**Study procedure**

All participants received oral supplementation with OMNi-BiOTiC^®^ FLORA plus at a dose of 2 × 2 g daily from the 15th to the 34th week of gestation. The probiotic preparation used in the study was OMNi-BiOTiC^®^ FLORA plus (Institut AllergoSan Pharmazeutische Produkte GmbH, Graz, Austria), administered orally at a dose of 2 × 2 g daily from the 15th to the 34th week of gestation. The product contains four Lactobacillus strains characteristic of a healthy vaginal microbiota: Lactobacillus crispatus LBV88, Lactobacillus rhamnosus LBV96, Lactobacillus gasseri LBV150N, and Lactobacillus jensenii LBV116. Each 2 g sachet provides a minimum of 5 × 10^9^ colony-forming units (CFU) of viable microorganisms.

The preparation is a synbiotic formulation combining probiotic strains with prebiotic components (maltodextrin and fructooligosaccharides), designed to support bacterial viability and activity. The product was supplied in individual sachets and stored by participants in accordance with the manufacturer’s instructions provided on the packaging. Importantly, the preparation does not require refrigeration, which facilitated storage and regular use throughout the study period.

Adherence to the supplementation protocol was actively monitored by the investigators throughout the study period. All participants remained under continuous obstetric care at the study centre for the duration of pregnancy, allowing for regular follow-up and direct contact. Compliance was assessed through scheduled visits as well as ongoing communication via telephone and e-mail, during which participants reported current use of the probiotic preparation, remaining sachets, and any potential interruptions.

Concomitant antibiotic therapy during probiotic supplementation was not permitted and constituted one of the predefined exclusion criteria. In the event that systemic antibiotic treatment had been required, probiotic supplementation would have been discontinued and the participant excluded from further analysis; however, no such cases occurred during the study. Participants were not instructed to modify their diet and did not use additional vaginal preparations, probiotics, or antiseptic agents during the supplementation period.

During the study, three vaginal and/or rectal swabswere taken:

1. At the time of study qualification (12th–15th week of pregnancy);
2. In the second trimester of pregnancy (22nd–25th week);
3. In the third trimester of pregnancy (weeks 34–35).

The collected biological material was subjected to standard microbiological diagnostics for the presence of *Streptococcus agalactiae* (GBS), in accordance with applicable laboratory procedures.

During each follow-up visit, the occurrence of any adverse eventsreported by participants or identified in the clinical trial was also monitored.

**Assessment of intervention effectiveness**

The efficacy of supplementation was assessed based on:

- The presence or absence of GBS colonisation in the third microbiological test (34th–35th week of pregnancy);
- The dynamics of changes in GBS colonisation at subsequent time points.

Analysis of the results from swabs taken in the second and third trimesters of pregnancy made it possible to determine the minimum time required to achieve a reduction or eradication of GBS carriageafter the start of supplementation.

## 3. Results

**Study population and screening outcomes**

During the recruitment period, 56 pregnant women underwent screening for Group B Streptococcus (GBS) colonisation as part of routine antenatal care, using vaginal and/or rectal swabs. A positive GBS result was identified in 10 of the 56 women (17.9%), who were subsequently enrolled in the intervention study. The remaining 46 women (82.1%) had negative screening results and were not included in further observation within the intervention arm.

For key proportions of negative GBS results at follow-up time points, 95% confidence intervals were calculated; however, due to the pilot nature of the study and the small sample size, the results are presented primarily in descriptive form.

**Characteristics of collected microbiological samples**

A total of 86 microbiological swabs were collected during the study period, including 56 screening swabs obtained at recruitment and 30 follow-up swabs collected from the 10 participants enrolled in the intervention study (three swabs per participant). All women included in the intervention arm had a positive GBS result in the qualifying swab (12–15 weeks of gestation), which constituted the inclusion criterion for further follow-up.

**GBS colonisation status at predefined follow-up time points**

At study qualification (first swab, 12–15 weeks of gestation), all participants (10/10; 100%) were colonised with GBS.

At the second sampling point (22–25 weeks of gestation), 5 of 10 participants (50%) tested negative for GBS, while the remaining 5 women (50%) continued to be colonised.

At the third sampling point (34–35 weeks of gestation), a negative GBS result was observed in 9 of 10 participants (90%), whereas in 1 participant (10%) the result remained positive, indicating persistent colonisation until the end of the observation period.

Detailed data on GBS colonisation status at successive time points are presented in Table 1.

### 3.1. Dynamics of GBS Colonisation Elimination

Analysis of individual participant trajectories demonstrated marked heterogeneity in the timing and pattern of GBS colonisation elimination during probiotic supplementation. Although all participants were GBS-positive at study entry, the course of colonisation differed substantially between individuals over the observation period.

In five participants (50%), the first negative GBS result was obtained already at the second trimester assessment (22–25 weeks of gestation), indicating early elimination of colonisation following initiation of supplementation. In this subgroup, clearance occurred within a relatively short interval after the start of probiotic administration. In contrast, in another four participants (40%), GBS colonisation persisted at the second time point, while a negative result was documented only at the third trimester assessment (34–35 weeks of gestation), reflecting delayed elimination during continued supplementation. In one participant (10%), GBS colonisation persisted at all assessed time points despite adherence to the supplementation protocol, representing a case of non-response within the study population.

**Figure 1:**
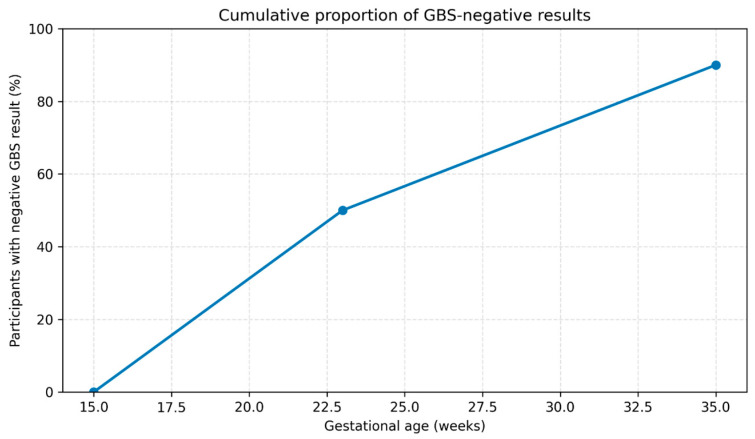
Cumulative proportion of GBS-negative results during probiotic supplementation. All participants were GBS-positive at baseline (15 weeks of gestation). The proportion of women with a negative GBS result increased progressively over time, reaching 50% in the second trimester (22–25 weeks of gestation) and 90% in the third trimester (34–35 weeks of gestation).

The time from initiation of supplementation (15 weeks of gestation) to the first documented negative GBS result ranged from 7.6 to 20.2 weeks. The median time to the first negative result was 8.6 weeks, while the mean time was 13.4 weeks, reflecting the presence of both early and delayed responders. The distribution of time to first negative GBS result exhibited a bimodal pattern, with one cluster of participants achieving clearance in the second trimester and a second cluster achieving clearance later in pregnancy. This distribution is illustrated in Figure 1.

### 3.2. Stability of GBS Colonisation Elimination

An important observation was the durability of GBS colonisation elimination once a negative result had been achieved. Among the nine participants who tested negative for GBS at the third sampling point (34–35 weeks of gestation), no recurrence of colonisation was observed during the remainder of the study period.

Specifically, no transitions from negative to positive GBS status were recorded between the second and third sampling points. This finding suggests that, within the limits of the observation period, elimination of GBS colonisation tended to be stable once achieved, without evidence of short-term recolonisation during ongoing supplementation.

### 3.3. Observational Data from the Non-Intervention Screened Population

To provide contextual information on the natural dynamics of GBS carriage later in pregnancy, additional observational data were analysed from women screened at the study centre who were not enrolled in the intervention arm. Among the 46 women who were GBS-negative at initial screening and did not receive probiotic supplementation, 14 (30.4%) tested positive for GBS at routine screening performed at 35–37 weeks of gestation.

This finding illustrates the variability of GBS colonisation status in late pregnancy in the absence of targeted microbiota-modulating intervention and highlights the potential for new colonisation to occur between early and late gestation.

### 3.4. Safety of Supplementation

Throughout the study period, supplementation with the multi-strain probiotic preparation was well tolerated. No adverse events related to probiotic use were reported, and none of the participants discontinued supplementation due to intolerance or side effects.

During scheduled follow-up visits and ongoing contact with the study team, participants did not report gastrointestinal symptoms or general complaints that could be attributed to the intervention. Furthermore, no cases of GBS re-colonisation were observed after a negative result had been obtained, and no safety concerns emerged during the observation period.

## 4. Discussion

Group B streptococcus (GBS) colonisation during pregnancy remains a significant clinical problem due to the risk of vertical transmission and severe neonatal infections. Despite the effectiveness of intrapartum antibiotic prophylaxis, this strategy does not affect GBS carriage during pregnancy and is associated with potential consequences in the form of microbiota disturbances and the development of antibiotic resistance. In this context, methods based on modulation of the vaginal microbiota are attracting increasing interest as a potential tool for reducing GBS colonisation before delivery.

**The significance of GBS carriage rates and the epidemiological context**

In this screening study, a positive GBS result was found in 17.9% of pregnant women, which is consistent with epidemiological data indicating that GBS carriage in the pregnant population ranges from 10 to 30% and in some populations may reach as high as 40% [15]. These differences are due, among other things, to geographical conditions, diagnostic methods and the timing of biological material collection. The frequency obtained confirms the representativeness of the study population and the validity of further analysis of the effectiveness of probiotic intervention.

**Reduction of GBS colonisation as a time-dependent process**

The results of this study indicate a gradual reduction in GBS colonisation during supplementation with a multi-strain probiotic preparation, with an increasing percentage of negative results at subsequent time points. The fact that in some participants colonisation was eliminated as early as the second trimester, while in others it was not until the third trimester, suggests individual differences in the susceptibility of the vaginal microbiota to modulation.

This distribution of responses is consistent with the concept that probiotic interventions do not work by directly eradicating the pathogen, but by gradually rebuilding the microbial ecosystem. This process may take time, especially in cases of initial vaginal dysbiosis or dominance of strains with lower protective potential.

**Vaginal microbiota as a key determinant of GBS persistence**

A growing body of evidence indicates that GBS colonisation is closely related to the structure of the vaginal microbiota. Microbiome studies have shown that in women with transient GBS colonisation in later stages of pregnancy, protective bacteria such as *Lactobacillus crispatus*, *Lactobacillus gasseri* and *Lactobacillus jensenii*, while in women with persistent colonisation, *Lactobacillus iners* predominates and the number *of L. crispatus* is reduced [81].

*Lactobacillus iners* is a strain commonly found in the vaginal microbiota, but its ability to maintain a stable, low pH and produce antimicrobial substances is limited compared to *L. crispatus*. Therefore, its dominance is sometimes considered a marker of an intermediate state between eubiosis and dysbiosis, favouring the colonisation of pathogens, including GBS [81].

**The importance of strain synergy in multi-strain preparations**

An important element in the interpretation of the results of this study is the use of a multi-strain preparation. Unlike the single-strain approach, multi-strain preparations have a more comprehensive effect on the microbiota, supporting several protective mechanisms simultaneously, such as competition for adhesion sites, lowering the pH of the vaginal environment, production of bacteriocins and modulation of the local immune response.

The results of the study by Starc et al. showed the protective effect of *Lactobacillus crispatus* dominance on GBS colonisation in the third trimester of pregnancy, but the authors emphasise that the effectiveness of this protection depends on the stability of the entire microbial ecosystem [85]. The data obtained in this study support this approach, indicating that the permanent elimination of GBS colonisation may be the result of the synergistic action of several strains of lactic acid bacteria, rather than the activity of a single microorganism.

**Durability of the effect and clinical significance**

An important observation was the stability of the effect achieved. Participants who tested negative for GBS in the third trimester did not show any re-colonisation by the end of the observation period. This may suggest that achieving a favourable vaginal microbiota profile promotes the maintenance of eubiosis and reduces the risk of pathogenic re-colonisation.

From a clinical point of view, the possibility of reducing GBS carriage before delivery is particularly important. Although intrapartum antibiotic prophylaxis remains the standard of care, microbiome interventions could in future reduce the number of women requiring antibiotic therapy, thereby reducing the exposure of newborns to antibiotics in the first hours of life.

**Safety of probiotic therapy during pregnancy**

The absence of adverse effects during supplementation and good tolerance of the preparation confirm the favourable safety profile of probiotics used in the study population. This is crucial in the context of pregnancy, where the safety of the intervention is the overriding criterion for eligibility for use.

**Limitations of the study**

This study has several limitations inherent to its pilot design. The small sample size, the single-arm structure, and the absence of a control group limit the strength of causal inference. In particular, Group B Streptococcus colonisation during pregnancy is a dynamic process, and spontaneous transitions from positive to negative status, as well as new colonisation later in pregnancy, may occur independently of any intervention. Therefore, it cannot be excluded that part of the observed reduction in GBS carriage reflects the natural course of colonisation rather than a direct effect of probiotic supplementation.

In addition, the lack of direct analysis of vaginal microbiota composition precludes a precise mechanistic interpretation linking clinical outcomes to specific microbiological changes. Consequently, the results should be interpreted as descriptive and hypothesis-generating rather than confirmatory. Furthermore, the absence of systematic combined vaginal and rectal sampling in all participants may have affected the sensitivity of GBS detection and limits direct comparability with studies employing standardised dual-site screening protocols.

**Directions for further research**

The results obtained justify further research involving a larger number of participants, preferably in a randomised design with a control group and using microbiome sequencing methods. This would allow for a more accurate assessment of the mechanisms of action of probiotics and their potential role in strategies for the prevention of GBS infections in pregnancy.

## 5. Conclusions

Group B streptococcus (GBS) carriage was found in 17.9% of pregnant women screened (which is within the range of frequencies described in population studies). Supplementation with a multi-strain probiotic preparation was associated with a gradual decrease in the frequency of GBS colonisation during pregnancy. The percentage of women with negative GBS results increased from 0% at the time of qualification to 50% in the second trimester and to 90% in the third trimester of pregnancy. The elimination of GBS colonisation showed varied dynamics, including both early and delayed responses to the intervention. In women who tested negative for GBS in the third trimester, no re-colonisation was observed until the end of the observation period. Probiotic supplementation was well tolerated and was not associated with any adverse effects. The results confirm the validity of further research into the role of vaginal microbiota modulation in reducing GBS carriage during pregnancy.

## Figures and Tables

**Table 1:** Group B streptococcus (GBS) colonisation status at successive time points during the study.

Time Point	Week of Pregnancy	Number of Participants (n)	GBS Negative, n (%)	GBS Positive, n (%)
First swab (qualification)	12–15 weeks	10	0	10 (100%)
Second smear	22–25 weeks	10	5 (50%)	5 (50%)
Third smear	34–35 weeks	10	9 (90%)	1 (10%)

## Data Availability

Data available after reasonable request from the corresponding author. During the preparation of this manuscript, the authors used Generative AI tools to assist in the creation of graphical figures, presentation of statistical analyses, and language refinement of the Discussion section. The authors reviewed and edited all outputs and take full responsibility for the content of this publication.
